# Frequency and Awareness of Risk Factors of Non-Communicable Diseases among University Students in Saudi Arabia

**DOI:** 10.12669/pjms.36.4.2400

**Published:** 2020

**Authors:** Mohamudha Parveen Rahamathulla, Mohemmed Sha M

**Affiliations:** 1 Dr. Mohamudha Parveen Rahamathulla, PhD. Department of Medical Lab Sciences, College of Applied Medical Sciences, Prince Sattam bin Abdulaziz University, Wadi Al Dawaser-11991, Kingdom of Saudi Arabia; 2 Dr. Mohemmed Sha M, PhD. Department of Computer Sciences, College of Arts & Sciences, Prince Sattam bin Abdulaziz University, Wadi Al Dawaser-11991, Kingdom of Saudi Arabia

**Keywords:** Knowledge, Lifestyle practices, Non-communicable diseases, Prevention, Saudi Arabia, University students

## Abstract

**Background and Objective:**

Non-communicable diseases (NCDs) are the leading cause of morbidity and mortality in developed countries. This study has evaluated the frequency of the risk factors of NCDs and its awareness among students in a University in Saudi Arabia.

**Methods:**

This cross-sectional study was conducted among 374 female students of Prince Sattam bin Abdulaziz University in Wadi Al Dawaser, Saudi Arabia. The study was carried out for a duration of six months, from August 2019 to January 2020. Standard self-administered questionnaire, anthropometric and biochemical parameters were used for data collection, analysed through SPSS version 20.0.

**Results:**

The mean age of the students was 20.6 years. The results showed that 64.7% of students were physically inactive, 52.4% spend more than two hours watching TV. The intake of adequate amount of fruits (14%) and vegetables (6.8%) was very little.. Junk food was consumed >11 times per week by 37.1%. The prevalence of overweight and severe obesity was 25.9% and 5.1% respectively. Blood sugar analysis showed 1.1% had pre-diabetes and 0.8% had diabetes. About 56.2% of students had no knowledge about NCDs and their risk factors.

**Conclusion:**

A high prevalence of risk factors for NCDs was found among students. Awareness programs about healthy lifestyle practices; periodic screening of school and college students at regular intervals with appropriate advice are warranted to control the rising epidemic of NCDs.

## INTRODUCTION

Non-communicable diseases (NCDs), also known as chronic diseases are generally of long duration and slowly progressive. Major types of NCDs are cardiovascular diseases, non-insulin dependent diabetes mellitus, osteoporosis, obesity, cancers, hypertension, etc.^1^ The incidence of NCDs is increasing globally and they pose a major public health concern. As per the World Health Organization (WHO), by 2020 it is estimated that NCDs will account for 73% of all deaths.^2^ Globally various sectors of the government and non-governmental organizations have been working with greater efforts in health promotion activities and improving the healthy lifestyle of their citizens.^3^

Over the last three decades, in the Kingdom of Saudi Arabia (KSA) there is a decline in communicable diseases but on the other way around, increase in NCDs.^4^ Ministry of Health (MoH) in the Kingdom provides free health care to its citizens.^5^ According to MOH, 65% of health care costs are being used for the treatment of these NCDs. The Government is paying much consideration to reduce this health style-related disease, as “Prevention is better than cure”. These diseases were given high priority the KSA’s health plans and strategies, as part of the comprehensive national development framework.^6^

However, in analysing the literature, there exist only a few studies^4^-^9^ in KSA describing data about NCDs and its risk factors among university students. Hence, this study was undertaken to assess the frequency of risk factors of NCDs and its knowledge among University students.

## METHODS

This cross-sectional study was conducted among female students of Prince Sattam bin Abdulaziz University in Wadi Al Dawaser, Eastern Saudi Arabia. The study was carried out for a duration of six months, from August 2019 to January 2020. A random convenient sampling of female students studying at different levels in the College of Arts & Sciences and Medical Sciences were involved. Students not willing to participate, reported to have any clinically diagnosed illness, on medication or pregnant were excluded.

A standard self-reported questionnaire was given to each student in Arabic language for data collection. The questionnaire was pre-tested randomly on 25 students beyond the sample size to ensure readability and proper administration of the data collection forms. The questionnaire was designed as per the Pan American Health Organization (PAHO) NCD surveillance Toolkit^10^, and WHO STEP-wise-approach-to-Surveillance (STEPS) of NCDs risk factors questionnaire^11^ with some minor modifications. The following data were collected;

### Socio-demographics details

It includes questions about age, marital status, study discipline, year of study, monthly family income, living with parents/spouse or alone and family history of chronic NCDs.

### Lifestyle and Dietary Habits

It includes questions about diet patterns, choice of foods, intake of fruits and vegetables, cigarette smoking, assessment of physical activity, hours spent in exercise, behavioural disorders, leisure activities, etc. The physical activity level was assessed based upon those who gave a positive response. They were further classified as those with adequate physical activity (≥150 min/week) based on the criteria of moderate intensity of minimum 30 minutes per day of their daily routine for at least five days a week.^11^ For fruits and vegetables intake 100 gm was considered as one serving. WHO recommends consumption of at least 400 grams (4 servings) of fruits and vegetables per day as adequate.^11^

### Anthropometrics Measurements

Student’s height, weight, hip and waist circumferences were measured. Body mass index (BMI) were calculated as, BMI = weight in kilograms divided by the height in square meters. The cut off values for BMI were based on WHO reference values.^11^ Waist circumference of >80 cm, Waist-hip ratio >0.85 for female was taken as abnormal.^11^ Blood Pressure was measured using the Omron digital BP apparatus. Three measurements of Systolic and diastolic BP were taken and the average was recorded. They were classified as follows: Normal BP (systolic < 120 mm Hg and diastolic < 80 mm Hg); prehypertension (systolic >120 to 139 mm Hg and diastolic >80 to 90 mm Hg) Stage-I hypertension (systolic >140 to 159 mm Hg and diastolic > 90 to 99 mm Hg); Stage-II hypertension (systolic blood pressure was > 160 mm Hg and diastolic > 100 mm Hg).^11^ Haemoglobin levels were measured using the Mission Hb Portable Haemoglobin meter, and the results were recorded accordingly. Random blood glucose levels were measured using ACCU-CHECK digital apparatus. They were classified as normal if <140 mg/dL and abnormal if >140 mg/dL as per the standard guidelines.^12^ The student above the normal range for random blood glucose level was subjected to fasting blood glucose testing upon their willingness. Fasting blood glucose was measured by an automated chemistry analyzer (Cobas 6000/C501). Fasting blood glucose concentration (Normal <100 mg/dL; pre-diabetes >100 to 125 mg/dL and diabetes >126 mg/dL) were considered as per the standard range.^12^

### Knowledge assessment about NCDs

Student’s knowledge and awareness about healthy lifestyle practices and NCDs risk factors were assessed. Questions included yes or no response patterns and others include Likertscale with a five-point response pattern.

### Ethical approval

Institutional Ethical approval (602/278) was obtained for the study. Verbal and written consent was obtained from every student. Statistical Package for Social Science (SPSS) version 20.0 was used for data analysis. Chi-Square test was applied to test the association among the variables and p-value < 0.05 was considered statistically significant with a 95% confidence level.

## RESULTS

A total of 374 undergraduate female students participated in the study. Socio-demographic details of the students are shown in Table-I. The mean age of the participants was 20.6±1.26 years and 316(84.5%) of them were unmarried. Of them 167(44.7%) were from the College of Science, 116(31.0%) from the College of Arts. About 341(91.2%) students were living with their families. Family history of NCDs data showed that 168(44.9%) participants reported as having no history. Positive family history of 47(12.6%) diabetes, 33(8.9%) obesity and 28(7.4%) hypertension cases were present (Table-I).

**Table-I:** Distribution of University Students according to their Socio-demographic characteristics (n=374).

Variables	University Students	

	Frequency (n)	Percentage (%)
***Age:***		
18-23	342	91.4
24-28	32	8.6
***Study Discipline:***		
Arts	116	31.0
Science	167	44.7
Medical Sciences	91	24.3
***Year of Study:***		
First	95	25.4
Second	132	35.3
Third	114	30.5
Fourth	33	8.8
***Marital Status:***		
Single	316	84.5
Married	58	15.5
***Monthly Family Income:***		
<5000 Riyals	51	13.6
5000-10000 Riyals	98	26.2
>10000 Riyals	225	60.2
***Residence Status:***		
Living with family	341	91.2
Living with friends	23	6.1
Alone	10	2.7
***Family History of Non-Communicable Diseases:***		
No History	168	44.9
History of Diabetes	47	12.6
History of Obesity	33	8.9
History of Hypertension	28	7.4
History of both diabetes and obesity	29	7.8
History of diabetes and hypertension	22	5.9
History of obesity and hypertension	23	6.1
History of diabetes, hypertension and obesity	11	2.9
History of Cardio Vascular Diseases	13	3.5

The physical activities and dietary habits of the participants are shown in Table-II. A high frequency of 242(64.7%) of physical inactivity was noted. About 132 (35.3%) students reported as being physically active. However, the time spent in physical activity was inadequate for 86(65.2%) students.

**Table-II:** Distribution of University Students according to their Physical activity and Dietary habits.

Characteristics	University Students	

	Frequency (n)	Percentage (%)
***Physical Activity:***		
Yes	132	35.3
No	242	64.7
***Time Spent in Physical Activity (n=132):***		
Adequate (≥150 min/week)	46	34.8
Inadequate (< 150 min/week)	86	65.2
***Nature of Physical activity (n=132):***		
Exercise/Sports Activity	33	25.0
Swimming	3	2.3
Gardening	47	35.6
Household works & others	49	37.1
***No. of hours spent in watching TV (per day):***		
<15 Minutes	54	14.4
15-30 Minutes	49	13.1
1-2 hrs	75	20.1
>2 hrs	196	52.4
***No. of hours spent in using Computer (per day):***		
<15 Minutes	27	7.2
15-30 Minutes	83	22.2
1-2 Hours	91	24.3
>2 Hours	173	46.3
***Daily Fruit Consumption:***		
Yes	209	55.9
No	165	44.1
***Fruits intake, servings per day (n=209):***		
1	82	39.2
2-3	51	24.4
3-4	47	22.4
>4	29	14.0
***Daily Vegetable Consumption:***		
Yes	162	43.3
No	212	56.7
***Vegetable intake, servings per day (n=162):***		
1	83	51.2
2-3	42	26.0
3-4	26	16.0
>4	11	6.8
***Cigarette Smoking:***		
Smoker	7	1.9
Non-smoker	367	98.1
***Consumption of Junk/Fast Food per week:***		
1-5 times	37	9.9
6-10 times	198	53.0
> 11 times	139	37.1

About 209 (55.9%) students reported consuming fruits daily. However, only 29 (14.0%) of them were consuming the recommended >4 servings of fruits per day. The choice of food was non-vegetarian for all the students. Similarly, 100% reported to consume junk food. Of them, 139 (37.1%) reported consuming junk food >11 times per week (Table-II). Nearly, 173 (46.3%) and 196 (52.4%) students were spending more than two hours per day using computers and watching TV respectively. Result analysis showed that 7(1.9%) students were smokers.

The anthropometrics and biochemical parameters of the participants are shown in Table-III. According to the computed BMI values, 97 (25.9%) were overweight and 19 (5.1%) had severe obesity. Around 113 (30.2%) students had abnormal waist circumference and 102 (27.3%) had abnormal waist-hip ratio.

**Table-III:** Distribution of study population according to anthropometrics and Biochemical parameters, (n=374).

Characteristics	University Students		Mean value ± SD

	Frequency(n)	Percentage(%)	
***Body Mass Index (Kg/m^2^):***			
Underweight (<18.5)	21	5.6	24.75±1.65
Normal (18.5 – 24.9)	185	49.5	
Overweight (25.0-29.9)	97	25.9	
Mild Obesity (30.0-34.9)	52	13.9	
Severe Obesity (≥35)	19	5.1	
***Waist Circumference (cm):***			
Normal (≤80)	261	69.7	78.49±0.95
Abnormal (>80)	113	30.2	
***Waist-Hip ratio:***			
Normal (≤ 0.85)	272	72.7	0.83±0.0095
Abnormal (>0.85)	102	27.3	
***Systolic Blood Pressure (mmHg):***			
Normal (<120)	241	64.4	123.29±2.93
Pre hypertension (120-139)	101	27.0	
Stage 1 hypertension (140-159)	32	8.6	
Stage 2 hypertension (≥160)	0	0.0	
***Diastolic Blood Pressure (mmHg):***			
Normal (<80)	265	70.8	79.83±1.67
Pre hypertension (80-89)	93	24.9	
Stage 1 hypertension (90-99)	16	4.3	
Stage 2 hypertension (≥100)	0	0.0	
***Random Blood glucose (mg/dL):***			
Normal (<140)	335	89.6	123.75±4.25
Abnormal (≥140)	39	10.4	
***Haemoglobin (mg/dL):***			
<11	33	8.8	12.69±0.29
11-11.9	105	28.1	
12-12.9	140	37.4	
13-15.9	96	25.7	

Analysis of the BP value shows that 101(27%) had systolic pre hypertension, 32 (8.6%) had systolic Stage-1 hypertension and none had Stage-2 hypertension. Random blood glucose level was normal for 335 (89.6%) and abnormal for 39 (10.4%) students. Of the 39 students tested for fasting blood sugar level, 32(82.1%) were normal and 4 (10.2%) had early diabetes and 3 (7.7%) had diabetes. Over all, of the total 374 study participants, 1.1% had pre-diabetes and 0.8% had diabetes.

**Fig.1:**
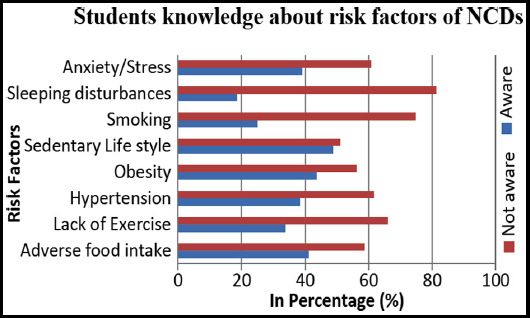
Students knowledge and awareness about Non-Communicable Diseases (NCDs) and its risk factors.

In assessing the student’s knowledge, 210 (56.2%) had no knowledge about NCDs and 126 (33.7%) students reported that NCDs are unpreventable. As shown in Fig.1, in analysing student’s awareness about the risk factors associated with NCDs, only 41.3% are aware of the adverse intake of foods. Nearly, 51.1% and 60.8% were reported to be unaware of sedentary lifestyle and stress respectively as the risk factors.

## DISCUSSION

In the present study majority of the students, 64.7% were physically inactive. Even students (n=132) who reported to perform physical activity, the time spent was not adequate as recommended for 65.2% of them. Further, 72.7% of students were interested only in doing gardening or household works rather than exercise or sports which may lead to inadequacy. In a national survey conducted in 2013, showed that 60% of Saudis were physically inactive.^13^ Our study results revealed a high prevalence of physical inactivity among female students are comparable with previous findings.^13^,^14^

This could be due to the fact that women cannot freely step out on their own or alone even for short distances and should always depend on cars. In addition, the culture and climatic conditions in Saudi Arabia also play a major reason. Further, the lack of indoor games and sports/gym activities for female students is a major drawback. In the present study, 52.4% and 46.3% of students reported to watch TV and using the computer respectively for more than two hours per day. This sedentary lifestyle behaviour among Saudi young generation is an alarming health threat. Our findings are higher than a study from Taif region and lower than studies from Riyadh and Jeddah.^9^,^15^

In the present study, 1.9% of students were reported to be smokers (Table-II). Different studies on smoking habits among female students in KSA have reported a prevalence rate of 1-6.8%.^9^,^13^ Our findings are in accordance with them; however, the low prevalence rate recorded in this study could be due to the fact that female students may underreport to be a smoker as smoking is considered as taboo in Saudi Community, especially in a rural region like Wadi Al Dawaser.

Analysis of the dietary habits among students showed that the adequate intake of vegetables was noticed in only 6.8% of them. This could be because the majority of the Saudi dishes like Kabsa, Mandi, Khuboos, etc. contains fewer or no vegetables. Further, University students largely depend on the college cafeteria for breakfast and lunch wherein all the items available are junk and fast foods leaving them without any healthy options.

Our study findings on BMI showed that 25.9% of students were overweight. Nearly, 13.9% and 5.1% were mild and severely obese respectively. Similar results were observed from recent studies done where 13.9% and 18.1% were obese. 9,14 Further, in the present work, 30.2% of the students had abnormal waist circumference and 27.3% of them had abnormal waist-hip ratio of >0.85 indicating central obesity. These findings are higher compared to other studies done among Saudi students.^9^,^15^ These high figures of waist circumference and obesity are due to the unhealthy food nature of the students. This study finding showed that physical inactivity is the leading factor of obesity in young adults. A significant negative correlation was found between BMI and the minutes of physical activity done (data not shown).

In the present study, systolic hypertension stage I was found in 8.6% of students and diastolic hypertension stage I was found in 4.3% of the students. This result is slightly lower than a Saudi National survey, where 12.5% of the population of adults were hypertensive.^14^ Further fasting blood glucose level analysis showed that 1.1% were pre-diabetic and 0.8% as diabetic. This result is lower compared to a recent report in 2019 from Riyadh where 3.8% female students were diabetic.^16^

The overall knowledge about NCDs and lifestyle-practices amongst University students was very poor (43.8%). Despite this, a proportion of students had knowledge about sedentary lifestyle (48.9%), obesity (43.7%) and adverse food habits (41.3%) as major risk factors of NCDs. This could be due to the presence of NCDs history in some of their families. However, in comparison to knowledge, the proportion practicing the recommended adequate physical activity was only 34.8% (n = 46). This finding showed that irrespective of the awareness the proportion involved in adequate physical activity is low. This is in agreement with a previous study that physical activity declines markedly during adolescence.^17^

### Limitations of the study

The limitation of this study is the small sample size with only female students. In future, large scale studies including both male and female students should be carried out. The strength of this study is that it mainly focused on the rural population which is usually neglected in many research surveys. The results of this study will add to the growing body of literature describing associations between health behaviours which play important roles in mediating chronic disease risk. Since the University students represent a major segment of the young population in the Kingdom, the health habits of them are of special concern as they are the key factor that influences the individual risk for NCDs and other chronic disorders later in their adult life. Further, this study finding would provide valuable data that could be used by administrators and policymakers to design strategies prioritize to address these issues concerning students’ health. Many studies like this at various local levels are needed to better understand the current scenario in the Kingdom.

## CONCLUSION

In the present study a high prevalence of risk factors for NCDs such as lack of physical activity, sedentary lifestyle, unhealthy food habits, etc. was found among the University students which is an alarming call for interventions. Adaptation of healthy lifestyle practices at a young age is crucial to avoid the occurrence of NCDs in later adulthood. Knowledge of NCDs and its risk factors were very poor among the students. Educational and awareness programs about healthy lifestyle practices are warranted particularly among the school level and university students. Schools and colleges throughout the Kingdom should provide sports and gym facilities for female students to practice the same. Periodic screening of the students at regular intervals with appropriate advice is also needed. If these current behaviours are not reversed during this adolescent period, the burden will rise in the future.

### Authors’ Contributions

**MPR:** Designed Research, data collection, drafted manuscript and responsible for the integrity of the work.

**MS:** Contributed in Research design, statistical correlations, article editing.
